# Effects of Alternating Mild‐Cold and Hot Water Immersion on Lower‐Leg Blood Flow Following Immersion in Healthy Young Adults

**DOI:** 10.1111/sms.70284

**Published:** 2026-04-15

**Authors:** Atsuya Toya, Nanako Hayashi, Daiki Imai, Takahiro Matsutake, Yuta Suzuki, Hisayo Yokoyama, Kazunobu Okazaki

**Affiliations:** ^1^ Department of Environmental Physiology for Exercise Osaka Metropolitan University Graduate School of Medicine Osaka City Osaka Japan; ^2^ Research Center for Urban Health and Sports Osaka Metropolitan University Osaka City Osaka Japan; ^3^ Research Fellow of the Japan Society for the Promotion of Science Chiyoda‐Ku Tokyo Japan; ^4^ Center for Health Science Innovation Osaka Metropolitan University Osaka City Osaka Japan

**Keywords:** alternating immersion, lower leg blood flow, skin blood flow, tissue oxygenation

## Abstract

Shear stress–mediated increases in blood flow are key physiological stimuli that regulate vascular endothelial function. This study assessed whether alternating lower‐leg immersion in mild‐cold (25°C) and hot water (42°C), with longer durations and repeated cycles, induces greater increases in post‐immersion blood flow. Sixteen healthy adults (11 males, 5 females; age, 23 ± 6 years) completed four randomized trials involving unilateral immersion of the left lower leg in mild‐cold and hot water: 3 min each ×2 (3CH2), 6 min each ×1 (6CH1), 3 min each ×4 (3CH4), and 6 min each ×2 (6CH2). After immersion, skin blood flow (SkBF) was significantly higher in 6CH2 than that in 3CH2 (*p* < 0.05), and popliteal artery (PA) blood flow was higher in 6CH2 than that in all other trials (*p* < 0.05), with significant time × trial interactions (*p* < 0.001). The area under the curve (AUC) for post‐immersion SkBF was higher in 6CH2 than that in 3CH2 (*p* < 0.05), and PA AUC was greater in 6CH2 than that in all other trials (*p* < 0.05). Core temperature remained unchanged, and cardiovascular variables exhibited only minor changes within the resting physiological range across all trials. Therefore, alternating mild‐cold and hot water immersions with longer and repeated cycles significantly increased lower‐leg blood flow following immersion. This strategy may represent a safe and well‐tolerated local thermal intervention for increasing peripheral circulation, including in individuals unable to engage in exercise or tolerate whole‐body heating.

## 1 Introduction

Vascular endothelial dysfunction associated with a sedentary lifestyle is a primary contributor to cardiovascular disease development [1]. Lower limb arteries, specifically the popliteal artery (PA), are particularly vulnerable to endothelial dysfunction, atherosclerosis, and peripheral vascular disease because of physical inactivity or sedentary behavior [2, 3]. Blood flow–induced shear stress plays an important role in regulating vascular endothelial function [4, 5, 6]. Interrupting sedentary behavior with physical movement or exercise is therefore widely recognized as an effective strategy to increase blood flow and shear stress [1, 4, 7]. Whole‐body hot water immersion has also been proposed as a non‐exercise alternative to induce similar hemodynamic stimuli [8, 9, 10]; however, such approaches may impose substantial cardiovascular and thermoregulatory strain and may not be feasible for individuals with limited exercise capacity or reduced heat tolerance [11, 12, 13, 14].

In contrast to whole‐body heating, local thermal interventions such as limb heating or local hot water immersion can increase peripheral blood flow while minimizing systemic cardiovascular and thermoregulatory strain [8, 15, 16, 17, 18]. Previous studies have demonstrated that local heating of the lower limbs increases conduit artery blood flow and shear stress, and induces both macrovascular and microvascular vasodilatory responses in healthy young and older adults [15, 17, 18, 19]. Furthermore, local heating has been shown to improve indices of vascular function without marked elevations in core temperature, supporting its feasibility as a peripheral vascular stimulus [15, 18, 19]. These characteristics suggest that local thermal interventions may provide a practical means of eliciting peripheral vascular stimuli in individuals who are unable to tolerate exercise or whole‐body heating.

Acute local heating activates temperature‐sensitive mechanisms in the skin, rapidly inducing thermal hyperaemia, an exponential increase in skin blood flow (SkBF), and shear stress [16, 17]. Like exercise, local heating increases blood flow and shear rate, which are associated with nitric oxide (NO)–mediated vascular signaling and shear stress–dependent vascular regulation [1, 5, 7, 18, 20]. In humans, local heating at 42°C induces marked skin vasodilation and increases limb perfusion without substantial systemic cardiovascular strain [1, 5, 7, 9, 18, 20, 21]. However, many local heating protocols require relatively long heating durations (≥ 30 min) to sustain vascular responses, which may be accompanied by increases in respiratory rate, sweating, cardiac preload, and core temperature [15, 22]. These thermal and cardiovascular loads may pose challenges for individuals with impaired thermoregulatory capacity owing to dehydration, physical inactivity [13], aging [13, 18, 23, 24], or chronic disease [11, 14, 25], thereby increasing the risk of adverse cardiovascular events during heat stress. Accordingly, strategies that can elicit peripheral vascular responses while limiting excessive heat accumulation are of particular interest.

Building on the limitations of prolonged local heating, alternating hot and cold water immersion has been proposed as a strategy to modulate peripheral vascular responses through repeated thermal stimuli while limiting excessive heat accumulation [26]. By intermittently interrupting heating with brief cooling phases, contrast immersion may allow sufficient thermal stimulation of the peripheral vasculature while preventing progressive elevations in core temperature and systemic cardiovascular strain [16, 22]. Previous studies have suggested that such thermal cycling can induce transient fluctuations in SkBF and shear rate, potentially contributing to peripheral vascular stimulation without sustained thermal loading [17, 19]. However, the relative contribution of heating duration vs. the repetition of thermal cycles to post‐immersion peripheral blood flow remains unclear, particularly when mild cooling is used primarily to maintain thermal stability rather than to independently augment perfusion.

Therefore, the purpose of the present study was to determine whether different patterns of alternating mild‐cold and hot water immersion, involving varying heating durations and repetition cycles, differentially affect post‐immersion lower‐leg blood flow and shear rate while minimizing systemic cardiovascular and thermoregulatory strain in healthy young adults. We hypothesized that protocols incorporating sufficient heating duration while maintaining thermal stability would elicit greater and more sustained post‐immersion hyperaemia than shorter or less effectively balanced protocols.

## 2 Materials and Methods

### 2.1 Ethics Statement

This study adhered to the principles of the Declaration of Helsinki and was approved by the Institutional Review Board of our institution (Kokuki #202401). All participants provided written informed consent after receiving detailed verbal explanations of the experimental protocol. Participants were informed that their participation was voluntary and that they could withdraw from the study at any time without disadvantage or penalty.

### 2.2 Participants

This study recruited 16 active, healthy young adults (11 males, 5 females; age 23 ± 6 years; body mass 60.9 ± 10.2 kg; height 168 ± 9 cm; body mass index 21.6 ± 2.3 kg/m [2], means ± standard deviation [SD]). All participants were non‐smokers, normotensive, and had no known cardiovascular or metabolic diseases. Participants who habitually took any vasoactive medications were excluded from the study. To reduce the potential confounding effect of endogenous sex hormones on cardiovascular and thermoregulatory responses, female participants with regular menstrual cycles for > 6 months, without oral contraceptives, were tested during the early follicular phase [27]. Before visiting our laboratory, the participants were instructed to abstain from caffeine‐containing beverages, vigorous physical activity, and alcohol for at least 24 h. Because no prior data were available for post‐immersion PA blood flow responses to alternating mild‐cold/hot immersion, an a posteriori power analysis was performed using G*Power (version 3.1, Universität Düsseldorf, Germany) based on the primary outcome (post‐immersion PA blood flow). Assuming a within‐subjects repeated‐measures design, an effect size derived from the observed trial × time interaction, an α level of 0.05, and a correlation among repeated measures estimated from the data, the achieved statistical power exceeded 0.80, indicating that the sample size was sufficient to detect the main effects of interest in this study. Although both men and women were included, the present study was not sufficiently powered to examine sex‐specific differences. Nevertheless, potential biological sex differences cannot be fully excluded and should be considered when interpreting these findings.

### 2.3 Study Design

**FIGURE 1:**
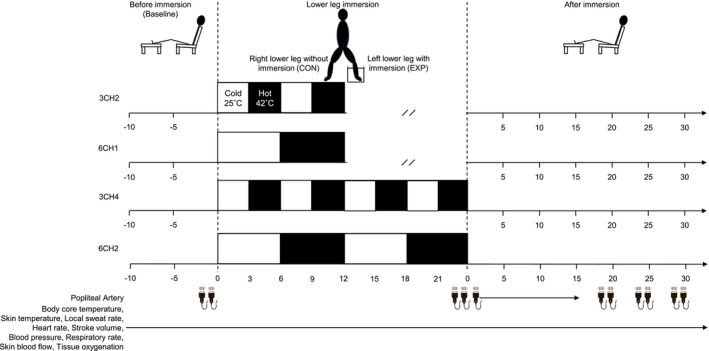
Experimental protocol.

A randomized crossover design was employed, and the order of the four immersion protocols was counterbalanced among the participants. Each experimental trial was separated by at least a 24‐h washout period. This interval was selected based on previous studies demonstrating that acute vascular and thermoregulatory responses to local heating and water immersion return to baseline within several hours after exposure [10, 15]. Although the 24‐h washout period was considered sufficient to minimize residual physiological effects between trials, the possibility of a small carryover effect cannot be entirely excluded and is acknowledged as a limitation of this study. The participants underwent four trials of different durations and numbers of cycles of mild‐cold (25°C) and hot (42°C) water immersion of the left lower leg: 3 min each ×2 (3CH2), 6 min each ×1 (6CH1), 3 min each ×4 (3CH4), and 6 min each ×2 (6CH2), on separate days (Figure 1). Each test was performed at the same time of day to avoid circadian variations. We used temperature gradients (8.5°C) from the normal lower leg skin temperature to induce heating and cooling on the skin surface. Additionally, 25°C was used for its minimal cardiovascular effects despite sufficient cool stimuli for vasoconstriction [22], whereas 42°C was used because it effectively increases SkBF and limb blood flow [23, 24, 28, 29]. Immersion durations of 3 and 6 min were used to determine the effects of an initial peak and subsequent increase in blood flow during heat‐induced vasodilation [30, 31]. The ratio of (1:1) for mild‐cold: Hot was applied for all trials to control the total immersion duration between 3CH2 and 6CH1, and 3CH4 and 6CH2.

### 2.4 Experimental Protocols

The participants were instructed to maintain a consistent diet between the trials and fast for at least 2 h before each trial. Body mass was measured while nude, after which the participants wore shirts and short pants. Experiments were performed in a climatic chamber (TBR‐6W2S2L2M; ESPEC Co., Osaka, Japan) maintained at 26.8°C ± 0.7°C (mean ± SD) with a relative humidity of 54.3% ± 6.4%. A finger cuff was placed on the second phalanx of the left index or middle finger to measure the blood pressure. Ear canal temperature (T_ear_) was measured using an infrared sensor inserted into the ear canal. A thermistor probe for measuring skin temperature (T_sk_) was positioned 13 ± 2 cm distal to the fibular head on both the experimental (EXP; left) and control (CNT; right) lower legs. SkBF and T_sk_ probes were placed adjacent to each other on the skin of both legs. A near‐infrared spectroscopy (NIRS) probe was used to measure the local tissue oxygenation profiles at the skin surface of the gastrocnemius lateral head at the EXP and CNT sites. Additionally, a capsule probe for the local sweat rate (LSR) was placed on the skin of the EXP site adjacent to the SkBF probe. Both probes were covered with a Tegaderm transparent film dressing (Tegaderm 1626; 3 M, London, Canada).

The experimental protocol is illustrated in Figure 1. The participants sat comfortably on a chair with their legs extended for at least 30 min during instrumentation. After baseline measurements had been recorded for 5 min, the PA was assessed using ultrasonography. The lower legs of the participants were then passively lowered, and the left leg was immersed in a 25°C water bath for 3 or 6 min, followed by immersion in 42°C hot water for 3 or 6 min. This procedure was repeated once, twice, or four times. The water temperature was controlled and maintained using water thermoregulators (Thermal Robo TR‐2α, AS ONE Co., Osaka, Japan). During all immersion protocols, the experimental limb was immersed at a standardized depth. The water level was maintained 5 cm distal to the fibular head, ensuring consistent immersion of the lower leg, while the knee joint and thigh remained out of the water. The participants were seated upright to maintain a consistent posture and hydrostatic pressure across all trials. After immersion, the lower legs were towel‐dried, extended, and passively returned to the baseline position. The participants rested for 30 min. During the experiment, the participants were constantly monitored by research personnel to minimize leg movements and avoid muscle contractions that could have affected the measurements. Variables were assessed before and after immersion, with the participants seated and their legs extended, and during immersion, with the participants seated and their lower legs lowered. After the removal of all measurement devices, the participants were weighed nude to assess total body water loss. The total duration of each immersion protocol was limited to a maximum of 12 min. Protocols involving longer continuous heating durations (e.g., a single 12‐min hot‐water immersion without intermittent cooling) were not included because prolonged local heating has been shown to progressively increase the core temperature and systemic cardiovascular strain. Given the aim of identifying a protocol that enhances peripheral vascular responses while maintaining thermal and cardiovascular stability, immersion durations exceeding 12 min of continuous heating were considered less suitable for this study. Mild‐cold water immersion (25°C) was incorporated between the heating phases as a thermal interval rather than as an independent stimulus to augment blood flow. This approach was intended to attenuate cumulative heat storage and prevent excessive elevations in core temperature during repeated heating cycles while allowing sufficient peripheral thermal stimulation during subsequent hot‐water immersion. Accordingly, the mild‐cold phase was designed primarily to maintain thermal stability and participant safety rather than to elicit vasoconstrictor‐driven hyperaemic responses.

### 2.5 Measurements

#### 2.5.1 Cardiovascular Variables

Heart rate (HR) was recorded using an electrocardiogram tracing (BSM‐7201; Nihon Kohden Co., Tokyo, Japan). Beat‐by‐beat blood pressure and stroke volume (SV) were measured using photoplethysmography with a cuff placed at the heart level on the second phalanx of the left middle finger (Finometer MIDI; Finapres Medical Systems BV, Enschede, The Netherlands). These variables were calculated from the pressure waveform using the Model Flow software that incorporates corrections for age, height, and weight (BeatScope 1.1; Finapres Medical Systems BV). The mean arterial pressure (MAP) was calculated using the diastolic pressure (DBP) and systolic pressure (SBP) as follows:

MAP=2/3×DBP+1/3×SBP

The local tissue oxygenation profiles of the gastrocnemius muscles were measured using NIRS (NIRO‐200NX; Hamamatsu Photonics KK, Hamamatsu, Japan). We assessed alterations in oxy‐hemoglobin (oxy‐Hb), deoxy‐hemoglobin (deoxy‐Hb), total‐hemoglobin (total‐Hb), and the tissue oxygenation index (TOI). Changes in total‐Hb were used as an index of the relative changes in local blood volume within the measured tissue. NIRS‐derived indices are commonly used to quantify within‐subject changes in oxygenation and relative blood volume during physiological and thermal interventions, and acceptable test–retest reproducibility has been reported for comparable NIRS measures [32]. These variables were assessed in 12 participants. Respiratory rate was measured using a CO_2_ monitor equipped with a nasal adapter (OLG‐2800; Nihon Kohden, Tokyo, Japan). The data were continuously recorded before, during, and after immersion. Baseline data were averaged for 5 min, and minute‐average data were calculated during and after immersion.

#### 2.5.2 Body Temperatures and Thermoregulatory Responses

T_ear_ was measured using an infrared sensor (Nipro CE Thermo; NIPRO Co., Osaka, Japan). The validity of T_ear_ as an index of body core temperature has been previously demonstrated by comparing it with esophageal temperature [33]. T_sk_ was measured using thermistors (ITP082‐24; Nikkiso‐Therm, Tokyo, Japan) placed on the skin of both lower legs. T_ear_ and T_sk_ data were collected every second using a data‐logger system (NT Logger Model N543; Nikkiso‐therm, Tokyo, Japan). SkBF at the EXP and CNT sites was measured using laser Doppler flowmetry (ALF21; Advance, Tokyo, Japan) and expressed as a percentage of the baseline mean value. Laser Doppler flowmetry has been widely used to assess relative changes in cutaneous perfusion during local thermal interventions, and its reproducibility for detecting within‐subject changes has been reported in previous methodological and experimental studies [34]. LSR was measured in the left lower leg using the ventilated capsule method (SKN‐2000; Nishizawa Electronic Measuring Instruments, Nagano, Japan). The data were recorded continuously before, during, and after immersion. Baseline data were averaged for 5 min, and the minute average data were calculated during and after immersion.

Thermal perception was measured based on thermal sensation and thermal comfort using perceptual scales at the end of baseline and immersion and every 5 min after immersion. The participants were asked to rate their thermal sensation on a scale ranging from −10 (very cold) to 10 (very hot), with 0 indicating neutrality. Additionally, they were asked to rate their overall comfort on a scale ranging from −10 (very uncomfortable) to 10 (very comfortable), with 0 indicating neutrality [35].

#### 2.5.3 PA Assessment

The PA was assessed in the left leg using a duplex ultrasound system (Vivid‐i; GE Healthcare, Tokyo, Japan) with a 12‐MHz linear probe, as described previously [1, 20, 36]. Measurements were performed at the end of baseline and immersion and every minute until 15 min after immersion, and then every 5 min until 30 min after immersion. PA assessments were performed by a trained investigator. The PA was imaged proximal to the bifurcation at or slightly above the popliteal fossa. To ensure consistent ultrasound measurements at the same point of the PA during and between trials, external (distance from the popliteal fossa) and internal landmarks (distance from the bifurcation) were used to guide accurate transducer placement. Ultrasound B‐mode variables were optimized to visualize the vessel walls in the longitudinal sections. The PA internal diameter was measured as the mean of three systolic and diastolic diameter measurements, with the mean diameter calculated as [23]:

$$\begin{array}{c} \text{Mean diameter}\;\left(\mathrm{mm}\right)\\ =\text{mean diastolic diameter}\times 2/3+\text{mean systolic diameter}\times 1/3 \end{array}$$

Pulsed‐wave Doppler was used to measure the blood velocity at a 60° insonation angle, with the sample volume encompassing the entire width of the artery. The blood flow velocity was averaged over 10–20 cardiac cycles. PA blood flow was calculated using the diameter and mean blood velocity as follows [17]:

$$\begin{array}{c} \text{Blood flow}\;\left(\mathrm{mL}/\min\right)=\pi \times {\left(\text{mean diameter}/2\right)}^{2}\\ \;\times \text{mean blood velocity}\times 60 \end{array}$$

The shear rate was calculated as follows:

$$\text{Shear rate}\;\left(s^{-1}\right)=4\times \text{mean blood velocity}/\text{mean diameter}$$

The vascular conductance of the PA was calculated as the ratio of PA blood flow to MAP corrected for the hydrostatic pressure difference between the heart level and the measurement site [37]. Local MAP was calculated as:

MAPlocal=MAPheart+0.78×height,

where height is the vertical distance (cm) between the heart level (sternal notch) and the measurement site (positive when below heart level). Vascular conductance was then expressed as:

Vascular conductance=PAblood flow/MAPlocal

The test–retest reliability of the ultrasound‐derived PA blood flow in the experimental leg was assessed using two baseline measurements obtained before each trial in all participants. The intraclass correlation coefficients (ICC (1, 2)) across all protocols ranged from 0.90 to 0.97, and the coefficients of variation (CV) for PA blood flow ranged from 6.2% to 9.3%, confirming high reproducibility of the measurements.

### 2.6 Statistics

Statistical analyses were performed using SigmaPlot version 15.5 (Systat Software Inc., San Jose, CA, USA). Data normality and homogeneity of variance were confirmed using the Shapiro–Wilk test and Brown–Forsythe test, respectively. For repeated‐measures ANOVA, the sphericity assumption was evaluated and, when violated, degrees of freedom were adjusted using the Greenhouse–Geisser correction. A two‐way (time × trial) repeated‐measures analysis of variance (ANOVA) was used to compare all variables before, at the end of, and after immersion across the trials. Alterations in SkBF, total‐Hb, oxy‐Hb, and PA blood flow from baseline after immersion for the 1–15 and 15–30 min intervals were quantified using the trapezoidal rule as the area under the curve (AUC). One‐way repeated‐measures ANOVA was used to compare AUC values across trials. Post hoc Bonferroni tests were conducted following significant primary effects or interactions. Values are presented as the mean ± SD. Statistical significance was set at *p* < 0.05. Effect sizes for main effects are reported as partial eta squared (ηp^2^), and 95% confidence intervals (CI) of the mean differences are provided, where applicable. Also, effect sizes for paired contrasts are reported as Cohen's dz [38].

## 3 Results

### 3.1 Cardiovascular Responses

The cardiovascular responses are summarized in Table 1. At the end of immersion, HR increased, and SV decreased by < 10% from baseline in all trials (*p* < 0.001), except for SV in 6CH1 (*p* = 0.445). No significant trial × time interactions or main effects of the trial were observed for the other cardiovascular variables. Changes over time were small and remained within the resting physiological range across all protocols.

**TABLE 1:** Cardiovascular variables at baseline, end of immersion, and after immersion.

		Immersion	After immersion (min)						*p*		
	Baseline	End	5	10	15	20	25	30	Time	Trial	Interaction
Heart rate, bpm											
3CH2	60 ± 8	65 ± 8*	60 ± 8	59 ± 8	59 ± 8	59 ± 9	61 ± 9	63 ± 9*	< 0.001	0.918	0.264
6CH1	60 ± 8	65 ± 8*	59 ± 8	60 ± 8	60 ± 7	60 ± 8	60 ± 8	61 ± 9			
3CH4	60 ± 8	66 ± 10*	60 ± 10	60 ± 9	59 ± 9	60 ± 8	61 ± 10	61 ± 8			
6CH2	59 ± 8	65 ± 8*	58 ± 7	58 ± 8	58 ± 8	60 ± 9	61 ± 9	61 ± 8			
Stroke volume, mL											
3CH2	74 ± 11	68 ± 11*	72 ± 11	73 ± 11	72 ± 11	72 ± 13	72 ± 13	70 ± 12	< 0.001	0.203	0.268
6CH1	74 ± 9	70 ± 9	74 ± 11	73 ± 11	74 ± 9	74 ± 10	74 ± 10	73 ± 10			
3CH4	78 ± 11	71 ± 14*	75 ± 11	77 ± 13	78 ± 13	77 ± 11	77 ± 12	76 ± 11			
6CH2	77 ± 13	72 ± 12*	76 ± 15	76 ± 14	77 ± 14	76 ± 14	75 ± 13	74 ± 13			
Cardiac output, L/min											
3CH2	4.4 ± 0.9	4.4 ± 1.0	4.3 ± 0.9	4.2 ± 0.9	4.3 ± 0.9	4.2 ± 1.0	4.3 ± 1.0	4.4 ± 1.1	< 0.001	0.262	0.998
6CH1	4.5 ± 0.8	4.6 ± 0.8	4.5 ± 0.9	4.4 ± 0.9	4.4 ± 0.8	4.4 ± 0.8	4.4 ± 0.9	4.5 ± 0.9			
3CH4	4.7 ± 0.7	4.6 ± 0.9	4.5 ± 0.7	4.4 ± 1.0	4.5 ± 0.7	4.6 ± 0.7	4.5 ± 1.0	4.6 ± 0.7			
6CH2	4.6 ± 1.0	4.7 ± 1.1	4.5 ± 1.1	4.4 ± 1.1	4.5 ± 1.0	4.5 ± 1.0	4.6 ± 1.0	4.5 ± 1.1			
Mean arterial pressure, mmHg											
3CH2	84 ± 9	81 ± 10	84 ± 9	84 ± 10	84 ± 9	84 ± 11	84 ± 10	84 ± 13	< 0.001	0.331	0.914
6CH1	86 ± 7	83 ± 6	87 ± 8	87 ± 7	87 ± 8	88 ± 9	87 ± 8	88 ± 10			
3CH4	86 ± 8	84 ± 10	87 ± 10	87 ± 10	87 ± 10	88 ± 9	88 ± 10	87 ± 9			
6CH2	85 ± 8	83 ± 9	86 ± 9	86 ± 10	86 ± 9	85 ± 10	85 ± 9	85 ± 10			
Respiratory rate, times/min											
3CH2	17 ± 2	18 ± 3	17 ± 2	17 ± 2	17 ± 2	17 ± 2	17 ± 2	18 ± 3	< 0.001	0.717	0.991
6CH1	17 ± 2	18 ± 3	17 ± 2	17 ± 3	17 ± 2	17 ± 3	17 ± 2	17 ± 2			
3CH4	17 ± 2	18 ± 3	17 ± 3	17 ± 3	17 ± 2	17 ± 2	17 ± 2	17 ± 2			
6CH2	17 ± 2	18 ± 2	17 ± 2	17 ± 2	16 ± 1	17 ± 1	17 ± 1	18 ± 2			

*Note:* Values are presented as means ± standard deviations. To simplify the table, one‐minute averages are presented at 5‐min intervals after immersion, with statistical analyses conducted on the complete dataset.

* Significant difference vs. baseline, *P* < 0.05.

### 3.2 Body Temperature and Thermoregulatory Responses

The body temperatures and thermoregulatory responses are presented in Table 2. T_ear_ remained unaltered throughout the experiment in all trials, indicating no increase in systemic thermal strain. T_sk_EXP_ increased from baseline at the end of immersion in all trials (*p* < 0.001) and was higher in 6CH1 and 6CH2 than in 3CH2 and 3CH4 (*p* < 0.001), with no difference between 6CH1 and 6CH2 or between 3CH2 and 3CH4. After immersion, T_sk_EXP_ gradually decreased but remained elevated above baseline across all trials (*p* < 0.001). T_sk_CNT_ decreased at the end of immersion (*p* < 0.001) and gradually returned toward baseline during recovery, although values remained slightly below baseline in all trials. Similarly, SkBF_CNT_ decreased at the end of immersion and recovered after immersion in all trials. Thermal sensation increased slightly at the end of immersion in all trials (*p* < 0.001) but returned to baseline thereafter, whereas thermal comfort remained unchanged throughout the experiment. The LSR in the lower leg increased minimally over time, with no differences among trials (time, *p* = 0.020; trial, *p* = 0.123; interaction, *p* = 0.801). Body mass loss was small and comparable among the trials (3CH2, 1.86 ± 0.90 g/min; 6CH1, 2.58 ± 1.55 g/min; 3CH4, 1.67 ± 0.56 g/min; and 6CH2, 1.91 ± 1.28 g/min; *p* = 0.079).

**TABLE 2:** Body temperatures and thermoregulatory responses at baseline, end of immersion, and after immersion.

		Immersion	After immersion (min)						*p*		
	Baseline	End	5	10	15	20	25	30	Time	Trial	Interaction
T_ear_ °C											
3CH2	36.63 ± 0.25	36.63 ± 0.28	36.62 ± 0.29	36.61 ± 0.27	36.61 ± 0.27	36.62 ± 0.28	36.62 ± 0.28	36.62 ± 0.27	0.855	0.845	0.967
6CH1	36.64 ± 0.22	36.63 ± 0.23	36.64 ± 0.24	36.65 ± 0.23	36.65 ± 0.23	36.64 ± 0.24	36.64 ± 0.23	36.64 ± 0.22			
3CH4	36.68 ± 0.19	36.67 ± 0.19	36.68 ± 0.19	36.67 ± 0.19	36.68 ± 0.18	36.68 ± 0.18	36.68 ± 0.18	36.67 ± 0.17			
6CH2	36.62 ± 0.23	36.67 ± 0.21	36.66 ± 0.20	36.67 ± 0.19	36.68 ± 0.18	36.67 ± 0.18	36.66 ± 0.18	36.65 ± 0.18			
T_sk_EXP_, °C											
3CH2	32.85 ± 0.43	39.52 ± 1.06*, ^b^, ^d^	35.24 ± 0.35*, ^d^	34.32 ± 0.34*, ^d^	33.99 ± 0.40*, ^d^	33.80 ± 0.45*, ^d^	33.67 ± 0.51*	33.58 ± 0.55*, ^d^	< 0.001	< 0.001	< 0.001
6CH1	32.79 ± 0.51	40.26 ± 0.63*, ^a^, ^c^	35.52 ± 0.48*	34.47 ± 0.57*	34.09 ± 0.66*	33.89 ± 0.71*	33.69 ± 0.78*	33.54 ± 0.82*, ^d^			
3CH4	32.84 ± 0.59	39.64 ± 0.92*, ^b^, ^d^	35.22 ± 0.36*, ^d^	34.35 ± 0.31*, ^d^	34.05 ± 0.37*	33.90 ± 0.44*	33.78 ± 0.51*	33.67 ± 0.52*			
6CH2	32.84 ± 0.61	40.57 ± 0.39*, ^a^, ^c^	35.79 ± 0.20*, ^a^, ^c^	34.77 ± 0.33*, ^a^, ^c^	34.43 ± 0.48*, ^a^	34.25 ± 0.55*, ^a^	34.08 ± 0.59*	34.01 ± 0.65*, ^a^, ^b^			
T_sk_CNT_, °C											
3CH2	32.50 ± 0.53	32.14 ± 0.52*, ^c^	32.23 ± 0.51*, ^c^	32.27 ± 0.50*, ^c^	32.28 ± 0.50*, ^c^	32.29 ± 0.52*	32.29 ± 0.55*	32.30 ± 0.59*	< 0.001	0.033	0.001
6CH1	32.45 ± 0.65	32.10 ± 0.82*	32.06 ± 0.62*	32.20 ± 0.69*	32.21 ± 0.71*	32.23 ± 0.74*	32.25 ± 0.78*	32.25 ± 0.82*			
3CH4	32.22 ± 0.47^d^	31.69 ± 0.54*, ^a^	31.78 ± 0.54*, ^a^	31.83 ± 0.55*, ^a^	31.85 ± 0.56*, ^a^	31.87 ± 0.59*	31.87 ± 0.62*	31.88 ± 0.65*			
6CH2	32.65 ± 0.82^c^	31.99 ± 0.57*	32.17 ± 0.73*	32.12 ± 0.67*	32.14 ± 0.73*	32.18 ± 0.77*	32.22 ± 0.82*	32.25 ± 0.88*			
SkBF__CNT_, %											
3CH2	100 ± 0	39 ± 28*	92 ± 32	99 ± 43	107 ± 42	114 ± 48	122 ± 69	115 ± 54	0.021	0.271	0.491
6CH1	100 ± 0	60 ± 27*	94 ± 26	96 ± 28	109 ± 40	101 ± 24	106 ± 28	102 ± 32			
3CH4	100 ± 0	53 ± 18*	91 ± 23	87 ± 21	84 ± 20	93 ± 27	85 ± 22^d^	85 ± 19			
6CH2	100 ± 0	64 ± 44*	102 ± 46	101 ± 37	104 ± 43	110 ± 50	123 ± 58^c^	121 ± 63			
Thermal sensation											
3CH2	1 ± 2	3 ± 1*	1 ± 1	1 ± 2	1 ± 2	1 ± 2	0 ± 2	1 ± 2	< 0.001	0.639	0.523
6CH1	0 ± 1	3 ± 2*	1 ± 1	1 ± 1	1 ± 2	1 ± 2	1 ± 2	1 ± 2			
3CH4	1 ± 1	3 ± 1*	1 ± 2	1 ± 2	1 ± 2	1 ± 2	0 ± 2	0 ± 1			
6CH2	1 ± 2	3 ± 2*	1 ± 1	1 ± 2	1 ± 2	1 ± 1	0 ± 2	0 ± 2			
Thermal comfort											
3CH2	2 ± 2	2 ± 3	2 ± 2	2 ± 2	2 ± 2	2 ± 2	2 ± 3	2 ± 2	0.054	0.284	0.288
6CH1	1 ± 2	3 ± 2	3 ± 2	2 ± 2	3 ± 2	3 ± 3	3 ± 2	2 ± 2			
3CH4	1 ± 2	3 ± 2	2 ± 2	2 ± 2	2 ± 2	1 ± 2	1 ± 2	1 ± 2			
6CH2	1 ± 2	2 ± 2	2 ± 2	2 ± 2	2 ± 2	2 ± 2	2 ± 2	2 ± 3			

*Note:* Values are presented as the mean ± standard deviation. T_ear_, ear‐canal temperature. T_sk_EXP_ and T_sk_CNT_ represent the skin temperatures in the experimental and control lower leg, respectively. SkBF_CNT_ represents the skin blood flow in the control lower leg. To simplify the table, one‐minute averages are presented at 5‐min intervals after immersion, with statistical analyses conducted on the complete dataset.

* Significant difference vs. baseline, *p* < 0.05.

^a^ Significant difference vs. 3CH2, *p* < 0.05.

^b^ Significant difference vs. 6CH1, *p* < 0.05.

^c^ Significant difference vs. 3CH4, *p* < 0.05.

^d^ Significant difference vs. 6CH2, *p* < 0.05.

### 3.3 SkBF, Tissue Oxygenation, and PA

**FIGURE 2:**
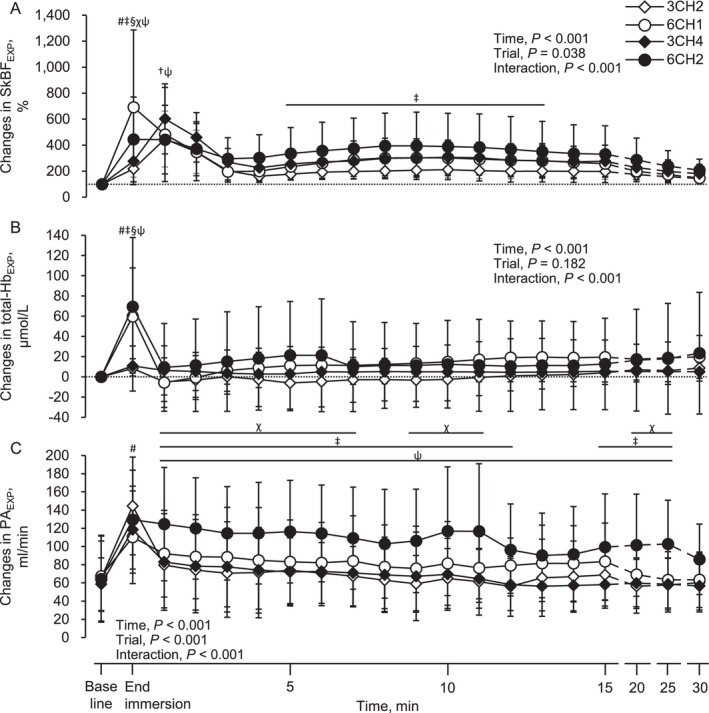
Alterations in skin blood flow (A), total‐hemoglobin (B), and popliteal artery blood flow (C) in the experimental lower leg at baseline, end of immersion, and after immersion. Values are presented as the mean ± standard deviation. SkBF_EXP_, total‐Hb_EXP_, and PA_EXP_ represent the skin blood flow, total‐hemoglobin, and popliteal artery blood flow in the experimental lower leg, respectively. #3CH2 vs. 6CH1, †3CH2 vs. 3CH4, ‡3CH2 vs. 6CH2, §6CH1 vs. 3CH4, χ6CH1 vs. 6CH2, and ψ3CH4 vs. 6CH2, *p* < 0.05. To simplify the figure, significant differences from baseline in each trial are not shown, but are described in the text.

Figure 2 illustrates the changes in SkBF_EXP_, total‐Hb_EXP_, and PA_EXP_. At the end of immersion, SkBF_EXP_ increased in all trials, except 3CH2; total‐Hb_EXP_ increased in 6CH1 and 6CH2, and PA_EXP_ increased in all trials (all, *p* < 0.001). SkBF_EXP_ was higher in 6CH1 than in the other trials and higher in 6CH2 than in 3CH2 and 3CH4. Total‐Hb_EXP_ was higher in 6CH1 and 6CH2 than in 3CH2 and 3CH4, whereas PA_EXP_ was higher in 3CH2 than in 6CH1. After immersion, SkBF_EXP_ increased above baseline during the early recovery period across all trials, with the most sustained elevation observed in 6CH2. Notably, SkBF_EXP_ in 6CH2 remained higher than that in 3CH2 and 3CH4 during the mid‐to‐late recovery phase. Total‐Hb_EXP_ showed a delayed post‐immersion increase in 6CH2, whereas no significant differences were detected between the trials. PA_EXP_ increased above baseline during the post‐immersion period, with the largest and most prolonged elevation observed in 6CH2 compared with the other trials.

Table 3 summarizes changes in the tissue oxygenation indices of the experimental lower leg. At the end of immersion, oxy‐Hb_EXP_ and deoxy‐Hb_EXP_ increased from baseline in 6CH1 and 6CH2 (*p* < 0.001). At this time point, oxy‐Hb_EXP_ was higher in 6CH2 than in 3CH2 and 3CH4. After immersion, oxy‐Hb_EXP_ increased above baseline in the mid‐to‐late post‐immersion period in 6CH1 and 6CH2, with higher values observed in 6CH2 than in 3CH4 at 30 min. TOI_EXP_ showed a modest decrease at the end of immersion, followed by a gradual recovery toward baseline across all trials. In the control lower leg, there was no significant effect of Trial in these indices (Table S1).

**TABLE 3:** Indices of tissue oxygenation in the experimental lower leg at baseline, end of immersion, and after immersion.

		Immersion	After immersion (min)						*p*		
	Baseline	End	5	10	15	20	25	30	Time	Trial	Interaction
Oxy‐Hb_EXP_, μmol/L											
3CH2	0.00 ± 0.00	−1.96 ± 16.17d	0.16 ± 14.51	4.23 ± 12.81	7.26 ± 10.19	8.54 ± 7.80	9.35 ± 10.82	12.99 ± 11.61	< 0.001	0.316	< 0.001
6CH1	0.00 ± 0.00	20.72 ± 20.92*	6.92 ± 19.64	13.11 ± 20.96	21.90 ± 20.97*	23.74 ± 21.36*	20.73 ± 19.94*	27.57 ± 25.42*			
3CH4	0.00 ± 0.00	2.90 ± 4.08^d^	2.88 ± 4.13	5.06 ± 6.17	5.85 ± 6.14	6.58 ± 6.27	6.56 ± 6.20	6.45 ± 5.25^d^			
6CH2	0.00 ± 0.00	34.17 ± 39.82*, ^a^, ^c^	13.51 ± 35.24	12.50 ± 30.12*	16.48 ± 33.70*	20.78 ± 38.06*	25.27 ± 42.75*	31.35 ± 45.24*, ^c^			
Deoxy‐Hb_EXP_, μmol/L											
3CH2	0.00 ± 0.00	9.87 ± 23.77^b^, ^d^	−6.16 ± 18.94	−6.81 ± 16.60	−3.08 ± 12.63	−1.31 ± 13.53	−3.09 ± 13.77	−4.29 ± 13.62	< 0.001	0.343	< 0.001
6CH1	0.00 ± 0.00	37.71 ± 33.11*, ^a^, ^c^	3.34 ± 9.91	0.86 ± 11.93	−3.24 ± 14.60	−6.95 ± 17.97	−2.91 ± 16.15	−1.66 ± 16.36			
3CH4	0.00 ± 0.00	7.17 ± 4.41^b^, ^d^	−0.70 ± 3.78	−0.79 ± 4.34	−1.15 ± 4.78	−1.96 ± 4.56	−2.10 ± 4.20	−2.13 ± 4.47			
6CH2	0.00 ± 0.00	35.15 ± 32.71*, ^a^, ^c^	8.45 ± 20.87	0.06 ± 14.54	−3.68 ± 14.67	−4.22 ± 15.70	−7.39 ± 18.22	−7.87 ± 22.95			
Total‐Hb_EXP_, μmol/L											
3CH2	0.00 ± 0.00	8.29 ± 30.18^b^, ^d^	−6.00 ± 26.81	−2.55 ± 21.04	4.17 ± 11.70	7.22 ± 11.67	6.25 ± 10.35	8.69 ± 13.72	< 0.001	0.182	< 0.001
6CH1	0.00 ± 0.00	59.80 ± 47.98*, ^a^, ^c^	11.15 ± 18.92	14.84 ± 16.68	19.54 ± 16.93	17.50 ± 14.84	18.90 ± 15.66	19.75 ± 21.25			
3CH4	0.00 ± 0.00	10.85 ± 7.10^b^, ^d^	2.99 ± 7.36	5.02 ± 9.97	5.52 ± 10.49	5.35 ± 10.33	5.21 ± 9.96	5.03 ± 9.20			
6CH2	0.00 ± 0.00	69.37 ± 68.46*, ^a^, ^c^	21.38 ± 53.21	12.56 ± 43.33	12.78 ± 45.23	16.55 ± 50.35*	17.91 ± 54.76*	23.47 ± 60.17*			
TOI_EXP_, %											
3CH2	67.81 ± 3.83	66.03 ± 6.24	68.79 ± 4.46	69.54 ± 4.38	68.94 ± 4.60	69.53 ± 4.99	69.76 ± 4.29	70.11 ± 4.65*	< 0.001	0.017	< 0.001
6CH1	65.88 ± 5.83	63.13 ± 7.39*, ^d^	65.03 ± 6.10^d^	65.78 ± 6.11^d^	66.75 ± 5.98^d^	67.50 ± 6.17	67.16 ± 6.75^d^	67.81 ± 6.47			
3CH4	68.14 ± 7.20	64.42 ± 7.77*	68.04 ± 6.60	68.99 ± 6.81	69.44 ± 6.97	70.26 ± 7.15	70.51 ± 7.16*	71.17 ± 7.33*			
6CH2	69.08 ± 4.25	67.84 ± 5.99^b^	69.46 ± 5.46^b^	70.48 ± 5.52^b^	70.97 ± 5.15*, ^b^	71.42 ± 5.35*	71.61 ± 4.67*, ^b^	71.48 ± 4.93*			

*Note:* Oxy‐Hb_EXP_, Deoxy‐Hb_EXP_, Total‐Hb_EXP_, and TOI_EXP_ represent oxy‐hemoglobin, deoxy‐hemoglobin, total‐hemoglobin, and tissue oxygenation index in the experimental lower leg, respectively. Values are presented as the mean ± standard deviation. To simplify the table, one‐minute averages are presented at 5‐min intervals after immersion, with statistical analyses conducted on the complete dataset.

* Significant difference vs. baseline, *p* < 0.05.

^a^ Significant difference vs. 3CH2, *p* < 0.05.

^b^ Significant difference vs. 6CH1, *p* < 0.05.

^c^ Significant difference vs. 3CH4, *p* < 0.05.

^d^ Significant difference vs. 6CH2, *p* < 0.05.

Table 4 summarizes alterations in PA in the experimental lower leg. The diameter remained unaltered throughout the experiment across all trials. In contrast, velocity, shear rate, and vascular conductance varied over time in parallel with changes in blood flow.

**TABLE 4:** Diameter, velocity, flow, shear rate, and conductance in the experimental lower leg popliteal artery at baseline, end of immersion, and after immersion.

		Immersion	After immersion (min)						*p*		
	Baseline	End	5	10	15	20	25	30	Time	Trial	Interaction
Diameter, mm											
3CH2	5.18 ± 0.72	5.24 ± 0.67	5.23 ± 0.68	5.17 ± 0.70	5.20 ± 0.70	5.18 ± 0.70	5.23 ± 0.73	5.16 ± 0.72	0.712	0.168	0.567
6CH1	5.30 ± 0.66	5.39 ± 0.71	5.37 ± 0.76	5.38 ± 0.74	5.32 ± 0.72	5.40 ± 0.72	5.34 ± 0.74	5.34 ± 0.73			
3CH4	5.33 ± 0.69	5.37 ± 0.65	5.22 ± 0.60	5.28 ± 0.60	5.29 ± 0.61	5.24 ± 0.59	5.26 ± 0.63	5.24 ± 0.62			
6CH2	5.32 ± 0.74	5.33 ± 0.71	5.29 ± 0.73	5.32 ± 0.70	5.35 ± 0.72	5.33 ± 0.70	5.26 ± 0.70	5.26 ± 0.72			
Velocity, cm/s											
3CH2	5.42 ± 4.00	10.79 ± 2.91*	5.81 ± 2.98^d^	5.31 ± 2.90^d^	5.46 ± 2.84	4.51 ± 2.59^d^	4.59 ± 2.49^d^	4.75 ± 2.39	< 0.001	< 0.001	< 0.001
6CH1	5.14 ± 2.95	8.21 ± 3.79*	6.33 ± 3.25^d^	6.15 ± 3.40^d^	6.47 ± 3.48	5.13 ± 2.61^d^	5.00 ± 2.62^d^	4.95 ± 2.64			
3CH4	4.44 ± 2.43	8.96 ± 3.59*	5.77 ± 3.08^d^	5.45 ± 3.51^d^	4.58 ± 2.45^d^	4.61 ± 2.08^d^	4.58 ± 2.26^d^	4.47 ± 2.33^d^			
6CH2	4.91 ± 3.63	9.94 ± 4.77*	8.98 ± 4.37*, ^a^, ^b^, ^c^	8.83 ± 5.43*, ^a^, ^b^, ^c^	7.49 ± 4.40*, ^c^	7.73 ± 4.51*, ^a^, ^b^, ^c^	8.23 ± 4.06*, ^a^, ^b^, ^c^	6.82 ± 3.30^c^			
Flow, ml/min											
3CH2	65.21 ± 47.13	144.55 ± 53.74*, ^b^	73.79 ± 37.95^d^	64.86 ± 32.90^d^	68.79 ± 34.21^d^	56.32 ± 29.58^d^	57.73 ± 29.76^d^	60.31 ± 32.23	< 0.001	< 0.001	< 0.001
6CH1	67.38 ± 38.63	110.16 ± 50.89*, ^a^	83.26 ± 45.93^d^	81.46 ± 45.96^d^	83.51 ± 42.24	69.20 ± 35.38^d^	63.33 ± 31.03^d^	63.46 ± 30.47			
3CH4	58.55 ± 28.93	118.51 ± 47.74*	72.10 ± 37.42^d^	69.41 ± 39.52^d^	58.12 ± 25.86^d^	59.71 ± 27.83^d^	58.62 ± 28.37^d^	56.75 ± 26.61^d^			
6CH2	64.19 ± 47.29	129.51 ± 54.34*	116.28 ± 56.38*, ^a^, ^b^, ^c^	116.80 ± 70.68*, ^a^, ^b^, ^c^	99.29 ± 58.52*, ^a^, ^c^	101.45 ± 56.14*, ^a^, ^b^, ^c^	102.76 ± 48.08*, ^a^, ^b^, ^c^	85.91 ± 38.71^c^			
Shear rate, s^−1^											
3CH2	43.65 ± 33.01	82.73 ± 22.59*	45.47 ± 23.84^d^	42.71 ± 25.99^d^	43.13 ± 24.43	35.81 ± 22.38^d^	36.24 ± 20.98^d^	37.38 ± 18.99	< 0.001	< 0.001	< 0.001
6CH1	39.61 ± 23.08	62.86 ± 30.73*	48.93 ± 25.19^d^	47.49 ± 27.95^d^	50.50 ± 29.04	39.23 ± 21.12^d^	39.43 ± 22.98^d^	38.69 ± 22.50			
3CH4	34.24 ± 20.67	68.91 ± 30.37*	45.56 ± 25.67^d^	42.63 ± 30.25^d^	35.82 ± 21.58^d^	35.86 ± 17.36^d^	35.88 ± 19.14^d^	35.11 ± 20.39^d^			
6CH2	37.93 ± 28.48	77.37 ± 43.34*	70.01 ± 35.47*, ^a^, ^b^, ^c^	67.86 ± 42.65*, ^a^, ^b^, ^c^	57.37 ± 34.00*, ^c^	59.41 ± 35.64*, ^a^, ^b^, ^c^	65.13 ± 33.72*, ^a^, ^b^, ^c^	53.71 ± 27.36^c^			
Conductance, unit											
3CH2	0.557 ± 0.418	1.277 ± 0.490*, ^b^, ^c^	0.627 ± 0.330^d^	0.550 ± 0.289^d^	0.580 ± 0.287^d^	0.472 ± 0.240^d^	0.488 ± 0.249^d^	0.502 ± 0.262	< 0.001	< 0.001	< 0.001
6CH1	0.562 ± 0.328	0.940 ± 0.444*, ^a^	0.692 ± 0.389^d^	0.669 ± 0.374^d^	0.687 ± 0.352	0.565 ± 0.291^d^	0.525 ± 0.257^d^	0.522 ± 0.252			
3CH4	0.482 ± 0.232	1.010 ± 0.408*, ^a^	0.604 ± 0.332^d^	0.583 ± 0.360^d^	0.486 ± 0.235^d^	0.485 ± 0.214^d^	0.483 ± 0.242^d^	0.464 ± 0.215^d^			
6CH2	0.539 ± 0.401	1.103 ± 0.457*	0.974 ± 0.474*, ^a^, ^b^, ^c^	0.973 ± 0.588*, ^a^, ^b^, ^c^	0.828 ± 0.481*, ^a^, ^c^	0.857 ± 0.485*, ^a^, ^b^, ^c^	0.871 ± 0.413*, ^a^, ^b^, ^c^	0.723 ± 0.331^c^			

*Note:* Values are presented as the mean ± standard deviation. To simplify the table, one‐minute averages are presented at 5‐min intervals after immersion, with statistical analyses conducted on the complete dataset.

* Significant difference vs. baseline, *p* < 0.05.

^a^ Significant difference vs. 3CH2, *p* < 0.05.

^b^ Significant difference vs. 6CH1, *p* < 0.05.

^c^ Significant difference vs. 3CH4, *p* < 0.05.

^d^ Significant difference vs. 6CH2, *p* < 0.05.

**FIGURE 3:**
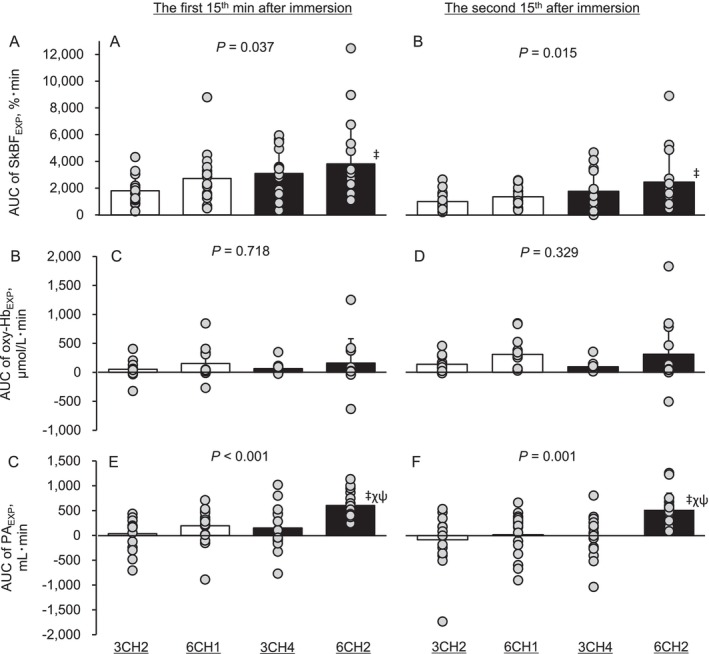
Area under the curve after immersion of skin blood flow (A and B), oxy‐hemoglobin (C and D), and popliteal artery blood flow (E and F) in the experimental lower leg at the end of immersion to 15 min after immersion (A, C, E), and between 15 and 30 min after immersion (B, D, F). Values are presented as the mean ± standard deviation (bar) with individual data (circles). SkBF_EXP_, oxy‐Hb_EXP_, and PA_EXP_ represent skin blood flow, oxy‐hemoglobin, and popliteal artery blood flow in the experimental lower leg, respectively.

Figure 3 illustrates the AUC of SkBF_EXP_, oxy‐Hb_EXP_, and PA_EXP_. A significant effect of trial was observed for the AUC of SkBF_EXP_ (ηp^2^ = 0.09 for the first and 0.11 for the second) and PA_EXP_ (ηp^2^ = 0.22 for the first and 0.19 for the second) in the first and second halves after immersion. The AUC of SkBF_EXP_ in 6CH2 was higher than that in 3CH2 (first: mean difference = 1877%·min, 95% confidence interval (CI) = [317, 3438], *p* = 0.030, dz. = 0.694; second: mean difference = 1399%·min, 95% CI = [257, 2541], *p* = 0.012, dz. = 0.693). The AUC of PA_EXP_ was highest in 6CH2, exceeding that in 3CH2 (first: mean difference = 569 mL·min, 95% CI = [360, 778], *p* < 0.001, dz. = 1.496; second: mean difference = 606 mL·min, 95% CI = [259, 954], *p* = 0.002, dz. = 0.959), 6CH1 (first: mean difference = 410 mL·min, 95% CI = [160, 659], *p* = 0.015, dz. = 0.904; second: mean difference = 502 mL·min, 95% CI = [167, 836], *p* = 0.015, dz. = 0.825), and 3CH4 (first: mean difference = 453 mL·min, 95% CI = [189, 716], *p* = 0.006, dz. = 0.945; second: mean difference = 521 mL·min, 95% CI = [211, 830], *p* = 0.011, dz. = 0.926).

## 4 Discussion

This study assessed whether alternating lower‐leg immersion in mild‐cold (25°C) and hot (42°C) water can increase post‐immersion blood flow, and whether these effects were dependent on immersion duration and cycle repetition. The results revealed that the 6CH2 protocol significantly increased both SkBF and PA blood flow compared with shorter or less repetitive protocols. Notably, the AUC for SkBF and PA blood flow post‐immersion was significantly higher in 6CH2 than in the other trials. No significant alterations were observed in the cardiovascular or thermal strain across the trials. Importantly, these findings demonstrate that post‐immersion peripheral hyperaemia can be augmented in the absence of meaningful systemic cardiovascular or thermoregulatory engagement. Rather than repetition of thermal cycles, the present results suggest that heating duration is the primary determinant of sustained post‐immersion blood flow.

The observed increase in SkBF and PA blood flow is likely mediated by a biphasic cutaneous vasodilatory response to local heating. The initial phase involves an axon reflex triggered by the thermal activation of sensory nerves, resulting in the release of vasodilator neuropeptides, such as substance P and calcitonin gene‐related peptide. This is followed by a sustained plateau phase mediated by NO and endothelium‐derived hyperpolarising factors [28]. However, comparison of protocols with identical total immersion durations (6CH2 vs. 3CH4) shows that the duration of individual heating bouts is more influential than the number of thermal transitions. Previous findings indicate that heat‐induced NO release—primarily through endothelial NO synthase (eNOS) activation—mediates sustained increases in skin blood flow following local warming, where eNOS inhibition significantly reduced cutaneous vasodilation [39]. Cyclic increases in blood flow during repeated hot‐immersion phases result in increased shear stress, a primary mechanical stimulus for endothelial adaptation. Repeated shear stress increased flow‐mediated dilation (FMD), increased NO bioavailability, and reduced oxidative stress [1, 4, 5]. Our findings of a significantly higher PA blood flow in the 6CH2 protocol are consistent with these mechanistic studies [1, 4, 5]. Additionally, transient shear increases have been associated with increased eNOS expression [6], indicating that shear levels similar to those induced by the 6CH2 protocol may activate the molecular mechanisms associated with vascular remodeling. An additional consideration is whether the greater post‐immersion blood flow observed in 6CH2 can be attributed solely to the final 6‐min heating bout. If this were the case, post‐immersion SkBF and PA blood flow responses would be expected to be comparable between 6CH1 and 6CH2, as both protocols have the same final heating duration. However, both the magnitude and duration of post‐immersion hyperaemia, as well as the AUC during recovery, were consistently greater in 6CH2 than in 6CH1, indicating that cumulative thermal exposure across the protocol, rather than the terminal heating phase alone, was a key contributor to the vascular response.

Although we did not directly measure endothelial function (e.g., via FMD or passive leg movement), the PA shear and blood flow responses observed may reflect mechanistic pathways relevant to vascular adaptation. Romero et al. demonstrated that the mean shear rate in the femoral artery increased by approximately 30–40 s^−1^ above baseline immediately after local heating, accompanied by an improvement in femoral FMD in older adults [18]. Similarly, Thomas et al. reported that, even 30 min after the cessation of local heating, the mean shear rate in the PA remained 10–20 s^−1^ higher than at baseline in older individuals [10]. In the present study, the mean shear rate in the 6CH2 protocol increased by approximately 70%–85% compared with baseline, which is largely consistent with the magnitude of shear elevation reported by Romero and Thomas [10, 18]. These hemodynamic responses may be relevant to mechanisms associated with endothelial maintenance in the lower limbs. Such localized vascular responses may be advantageous in individuals who cannot tolerate whole‐body heat therapy, highlighting the potential vascular benefits of localized heating. Nevertheless, direct assessments of endothelial function are required to confirm whether these hemodynamic responses translate into functional adaptation.

Despite a clear increase in SkBF, muscle oxygenation responses were limited; although oxy‐Hb and deoxy‐Hb increased at the end of immersion in the 6CH1 and 6CH2 trials, these changes were transient and did not translate into sustained or functionally meaningful alterations in muscle oxygenation (Table 3). This reduced response may reflect insufficient thermal penetration into the deeper musculature, specifically because of the absence of active muscle contraction and the limited penetration depth of surface heating. High baseline muscle oxygenation in healthy young adults may have limited the potential for measurable alterations. Previous studies using deeper thermal modalities (ultrasound and shortwave diathermy) or combining heating with exercise have reported significant increases in muscle oxygenation, which are effects that may not be replicated with surface heating alone [40]. Thus, alternating immersion appears to preferentially enhance superficial and conduit artery perfusion rather than deep muscle oxygenation under resting conditions.

Importantly, despite significant increases in peripheral blood flow, T_ear_ and blood pressure remained unchanged, and HR and SV showed small but significant transient changes at the end of immersion; however, these responses remained within the resting physiological range and returned toward baseline during recovery in all trials (Tables 1 and 2), indicating minimal systemic cardiovascular strain. These findings confirm that the increased peripheral blood flow is locally mediated and not driven by systemic cardiovascular engagement. This is consistent with the findings of Thomas et al. [10], who demonstrated that limb heating can significantly increase PA shear rate without altering cardiac output. The inclusion of mild‐cold phases likely limited excessive heat accumulation while preserving peripheral vascular stimulation. This localized hemodynamic effect is clinically significant in individuals who cannot tolerate systemic hyperthermia or high‐intensity activity.

In contrast to full‐body heating modalities or thermal suits that induce sweating, thermal discomfort, and cardiovascular strain, our results indicated that alternating immersion at moderate temperatures provides a thermally efficient and well‐tolerated method for increasing leg perfusion. Longer local heating (≥ 30 min) often results in thermal discomfort or cardiovascular strain, specifically in the elderly or clinical populations with impaired thermoregulatory capacity [22, 24]. The inclusion of cold phases during alternating immersion may attenuate thermal accumulation, reduce the excessive pooling of blood in the peripheral circulation, and increase overall participant tolerance. Additionally, the observed lack of thermal accumulation supports previous reports that contrast therapy may increase peripheral blood flow without significantly affecting core temperature [26].

## 5 Limitations

This study has several limitations. First, endothelial function was not directly assessed using gold‐standard methods, such as FMD or passive leg movement [4]. Although PA shear and blood flow are valuable surrogates, they do not confirm the underlying mechanisms [30, 39]. Second, the study included only healthy young adults, limiting its generalizability to older or clinical populations [11, 18]. Third, the lack of pharmacological blockade (NO synthase inhibition or sensory nerve ablation) prevents the definitive attribution of effects to specific pathways. Finally, this study focused on acute vascular responses; whether repeated exposure results in functional or structural adaptation remains to be determined. Future studies should explore the chronic effects of alternating immersion using longitudinal designs.

## 6 Conclusions

Alternating lower‐leg immersion in mild‐cold and hot water using longer heating bouts (6CH2) effectively increased both superficial and conduit artery blood flow. This protocol elicited robust local hyperaemic responses while avoiding systemic cardiovascular and thermal strain, highlighting heating duration—rather than repetition alone—as the key determinant of post‐immersion peripheral perfusion. The simplicity, safety, and tolerability of this approach support its potential utility as a localized strategy to augment lower‐limb blood flow.

## 7 Perspective

Since PA is specifically susceptible to dysfunction owing to longer sitting or inactivity, uninterrupted sitting results in impaired PA endothelial function because of reduced shear stress [41]. Approaches that increase lower‐limb shear stress may therefore be important for maintaining endothelial function. In this context, alternating mild‐cold and hot water immersion may represent a feasible adjunct strategy to transiently augment lower‐limb perfusion when exercise or whole‐body heating is contraindicated. Future studies should determine whether repeated exposure to this thermal protocol induces sustained improvements in endothelial function, vascular compliance, or perfusion reserve. In addition, examining dose–response relationships between heating duration and cycle repetition would help optimize intervention design. Including older adults and patients with type 2 diabetes and peripheral artery disease may increase the clinical applicability of the study. Additionally, combining the thermal stimulus with active muscle engagement (neuromuscular electrical stimulation or low‐level cycling) may increase deep tissue perfusion. Finally, mechanistic studies incorporating pharmacological inhibitors can help distinguish the contributions of NO, prostaglandins, and sensory nerve pathways.

## Author Contributions

A.T. and K.O. conceived and designed the study. A.T., N.H., D.I., T.M., Y.S., H.Y., and K.O. conducted the study. A.T. and K.O. analyzed and interpreted the data. A.T. prepared the tables and figures and drafted the first manuscript. A.T. and K.O. edited and revised the manuscript. All authors approved the final version of the manuscript.

## Funding

This work was supported by the Japan Society for the Promotion of Science (K24K02818).

## Ethics Statement

This study adhered to the Declaration of Helsinki and was approved by our institutional Ethics Committee (Kokuki #202401).

## Consent

All participants provided written informed consent after receiving detailed verbal explanations of the experimental protocol.

## Conflicts of Interest

The authors declare no conflicts of interest.

## Supporting information


**Table S1:** Indices of tissue oxygenation in the control lower leg at baseline, at the end of immersion, and after immersion. Oxy‐Hb_CNT_, Deoxy‐Hb_CNT_, Total‐Hb_CNT_, and TOI_CNT_ represent oxy‐hemoglobin, deoxy‐hemoglobin, total‐hemoglobin, and tissue oxygenation index in the experimental lower leg, respectively. Values are presented as the mean ± standard deviation. To simplify the table, one‐minute averages are presented at 5‐min intervals after immersion, with statistical analyses conducted on the complete dataset. *Significant difference vs. baseline, *p* < 0.05.

## Data Availability

The data that support the findings of this study are available on request from the corresponding author. The data are not publicly available due to privacy or ethical restrictions.
