# Supplementary material for: Effects of Alternating Mild‐Cold and Hot Water Immersion on Lower‐Leg Blood Flow Following Immersion in Healthy Young Adults
Source: Scand J Med Sci Sports. 2026 Apr 15;36(4):e70284. doi: 10.1111/sms.70284 (PMC13081763; doi:10.1111/sms.70284)
Supplement: Supplementary file 1 — Table S1: Indices of tissue oxygenation in the control lower leg at baseline, at the end of immersion, and after immersion. Oxy‐HbCNT, Deoxy‐HbCNT, Total‐HbCNT, and TOICNT represent oxy‐hemoglobin, deoxy‐hemoglobin, total‐hemoglobin, and tissue oxygenation index in the experimental lower leg, respectively. Values are presented as the mean ± standard deviation. To simplify the table, one‐minute averages are presented at 5‐min intervals after immersion, with statistical analyses conducted on the complete dataset. *Significant difference vs. baseline, p < 0.05. [file SMS-36-e70284-s001.docx]

**Supplement Table 1**. Indices of tissue oxygenation in the control lower leg at baseline, end of immersion, and after immersion.

|  |  | Immersion | After immersion (min) | | | | | | *P* values | | |
| --- | --- | --- | --- | --- | --- | --- | --- | --- | --- | --- | --- |
|  | Baseline | End | 5 | 10 | 15 | 20 | 25 | 30 | Time | Trial | Interaction |
| Oxy-Hb_CNT_, μmol/L |  |  |  |  |  |  |  |  |  |  |  |
| 3CH2 | 0.00±0.00 | -6.66±19.17 | -0.52±10.26 | 1.71±9.43 | 4.89±10.99 | 4.80±14.29 | 4.89±13.28 | 7.20±16.42 | < 0.001 | 0.689 | < 0.001 |
| 6CH1 | 0.00±0.00 | -12.98±33.44 | -9.88±33.60 | -6.37±33.87 | -0.68±33.85 | -3.14±31.41 | 7.99±7.85 | 3.41±34.84 |  |  |  |
| 3CH4 | 0.00±0.00 | 0.71±2.81 | -0.08±3.32 | 0.76±3.55 | 0.62±2.73 | 0.77±2.67 | 0.85±2.68 | 1.01±2.97 |  |  |  |
| 6CH2 | 0.00±0.00 | 6.67±31.87 | -11.87±22.12 | -8.67±20.63 | -6.87±18.79 | -2.98±18.46 | 1.59±20.53 | 2.15±16.06 |  |  |  |
| Deoxy-Hb_CNT_, μmol/L |  |  |  |  |  |  |  |  |  |  |  |
| 3CH2 | 0.00±0.00 | 35.75±29.62* | 1.78±11.60 | -0.41±12.10 | 1.63±12.60 | 0.99±16.16 | 0.32±14.80 | -0.57±14.20 | < 0.001 | 0.376 | < 0.001 |
| 6CH1 | 0.00±0.00 | 30.18±30.64* | -12.11±34.64 | -11.14±36.94 | -13.65±37.76 | -18.20±38.49 | -5.58±15.73 | -18.64±38.13 |  |  |  |
| 3CH4 | 0.00±0.00 | 9.26±5.04 | 0.11±3.94 | -0.22±4.03 | -0.22±4.21 | -0.48±4.05 | -0.46±3.58 | -0.13±3.98 |  |  |  |
| 6CH2 | 0.00±0.00 | 38.52±34.85* | -2.79±12.61 | -7.12±16.41 | -6.53±18.42 | -8.01±19.44 | -9.72±19.96 | -9.04±21.27 |  |  |  |
| Total-Hb_CNT_, μmol/L |  |  |  |  |  |  |  |  |  |  |  |
| 3CH2 | 0.00±0.00 | 29.07±24.03* | 1.12±14.66 | 1.28±14.01 | 6.47±11.63 | 5.60±10.47 | 6.49±13.21 | 7.23±15.62 | < 0.001 | 0.364 | < 0.001 |
| 6CH1 | 0.00±0.00 | 25.91±36.57* | -0.86±9.78 | 3.97±9.94 | 7.15±10.17 | 0.45±9.13 | 3.50±9.04 | 5.40±9.07 |  |  |  |
| 3CH4 | 0.00±0.00 | 10.03±6.84 | 0.06±6.32 | 0.58±6.84 | 0.44±6.32 | 0.33±6.12 | 0.43±5.83 | 0.91±6.19 |  |  |  |
| 6CH2 | 0.00±0.00 | 45.23±55.90* | -14.72±33.80 | -15.86±36.13 | -13.46±36.85 | -11.05±37.01 | -8.19±38.54 | -6.96±35.24 |  |  |  |
| TOI_CNT_, % |  |  |  |  |  |  |  |  |  |  |  |
| 3CH2 | 67.54±5.06 | 62.33±5.41* | 67.54±5.01 | 68.18±5.38 | 68.18±5.39 | 68.44±5.54 | 68.39±5.62 | 68.61±4.93 | < 0.001 | 0.222 | 0.720 |
| 6CH1 | 64.96±5.14 | 60.91±5.54* | 65.59±4.89 | 65.79±5.32 | 65.98±5.27 | 66.72±5.04* | 66.75±5.11 | 66.70±5.02* |  |  |  |
| 3CH4 | 68.44±5.89 | 63.82±6.61* | 68.09±5.59 | 68.62±5.82 | 68.54±5.82 | 68.64±5.91 | 68.59±6.18 | 68.81±6.10 |  |  |  |
| 6CH2 | 68.97±5.37 | 64.50±5.32* | 68.31±5.08 | 69.02±5.38 | 69.02±5.33 | 69.40±5.43 | 69.96±5.31 | 69.81±5.87 |  |  |  |

Oxy-Hb_CNT_, Deoxy-Hb_CNT_, Total-Hb_CNT_, and TOI_CNT_ represent oxy-hemoglobin, deoxy-hemoglobin, total-hemoglobin, and tissue oxygenation index in the experimental lower leg, respectively. Values are presented as the mean ± standard deviation. To simplify the table, one-minute averages are presented at 5-minute intervals after immersion, with statistical analyses conducted on the complete dataset. *Significant difference vs. baseline, *P* < 0.05.
